# Heat Treatments and Critical Quenching Rates in Additively Manufactured Al–Si–Mg Alloys

**DOI:** 10.3390/ma13030720

**Published:** 2020-02-05

**Authors:** Leonhard Hitzler, Stephan Hafenstein, Francisca Mendez Martin, Helmut Clemens, Enes Sert, Andreas Öchsner, Markus Merkel, Ewald Werner

**Affiliations:** 1 Institute of Materials Science and Mechanics of Materials, Technical University Munich, 85748 Garching, Germany; hafenstein@wkm.mw.tum.de (S.H.); werner@wkm.mw.tum.de (E.W.); 2 Department of Materials Science, Montanuniversität Leoben, 8700 Leoben, Austria; mendez@unileoben.ac.at (F.M.M.); helmut.clemens@unileoben.ac.at (H.C.); 3 Faculty of Mechanical Engineering, Esslingen University of Applied Sciences, 73728 Esslingen, Germany; enes.sert@hs-esslingen.de (E.S.); andreas.oechsner@hs-esslingen.de (A.Ö.); 4 Institute for Virtual Product Development, Aalen University of Applied Sciences, 73430 Aalen, Germany; markus.merkel@hs-aalen.de

**Keywords:** hardness, microstructure, grain morphology, silicon segregations, laser powder-bed fusion

## Abstract

Laser powder-bed fusion (LPBF) has significantly gained in importance and has become one of the major fabrication techniques within metal additive manufacturing. The fast cooling rates achieved in LPBF due to a relatively small melt pool on a much larger component or substrate, acting as heat sink, result in fine-grained microstructures and high oversaturation of alloying elements in the α-aluminum. Al–Si–Mg alloys thus can be effectively precipitation hardened. Moreover, the solidified material undergoes an intrinsic heat treatment, whilst the layers above are irradiated and the elevated temperature in the built chamber starts the clustering process of alloying elements directly after a scan track is fabricated. These silicon–magnesium clusters were observed with atom probe tomography in as-built samples. Similar beneficial clustering behavior at higher temperatures is known from the direct-aging approach in cast samples, whereby the artificial aging is performed immediately after solution annealing and quenching. Transferring this approach to LPBF samples as a possible post-heat treatment revealed that even after direct aging, the outstanding hardness of the as-built condition could, at best, be met, but for most instances it was significantly lower. Our investigations showed that LPBF Al–Si–Mg exhibited a high dependency on the quenching rate, which is significantly more pronounced than in cast reference samples, requiring two to three times higher quenching rate after solution annealing to yield similar hardness results. This suggests that due to the finer microstructure and the shorter diffusion path in Al–Si–Mg fabricated by LPBF, it is more challenging to achieve a metastable oversaturation necessary for precipitation hardening. This may be especially problematic in larger components.

## 1. Introduction

Various additive manufacturing methods for direct metal fabrication have emerged in the last decade, which can be used to fabricate directly deployable components without the necessity of a post-densification process [1,2]. Some of the most prominent representatives are the powder-bed fusion techniques, whereby a laser or electron beam is utilized to repetitively melt sections in a powder layer, resembling the sliced approximation of the component to be manufactured, and gradually, in a layer by layer approach, fabricate the entire component. Within this study, the vast field is narrowed down to laser powder-bed fusion (LPBF) of hypo-eutectic aluminum silicone magnesium (Al–Si–Mg) alloys.

**Figure 1:**
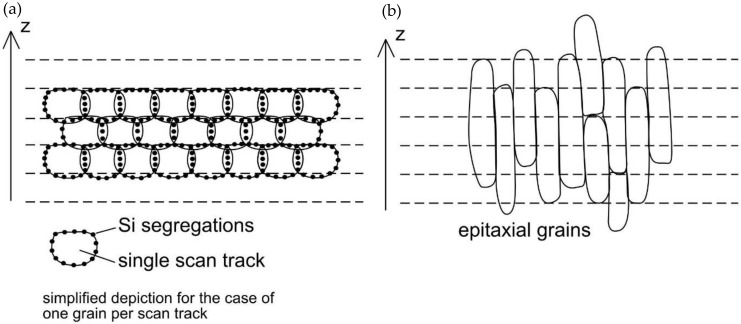
Simplified microstructural characteristics of (**a**) Al–Si–Mg and (**b**) stainless steel; taken from [5].

Al–Si–Mg alloys exhibit a unique microstructure in their as-fabricated state, whereby the macroscopic anisotropy characteristics are predominantly governed by the localized formation of silicon segregations; see Figure 1 [3,4]. This is in strong contrast to other alloys, like austenitic stainless steels or Inconel, with their dominating characteristic being their grain morphology [5,6]. The cause for the major impact of the Si-segregations is thought to be their location, as they occur most prevalently in remolten areas, i.e., in the bonding areas between single scan tracks and subsequent layers [7]. Compared to the aluminum solid solution (α-Al), the Si-segregations are brittle and prone to shear fracture [8]. Due to the layer-wise building approach in the LPBF process, these embrittlements repetitively occur every single layer and become the governing weakness, defining the observable macroscopic properties, as has been documented for tensile and compression strength as well as for the fracture toughness [8,9,10,11,12]. Even though these weaknesses are present, LPBF Al–Si–Mg alloys exhibit superior mechanical properties compared to their cast counterparts, at least under static loading, while the fatigue performance suffers from surface roughness and sub-surface defects [13,14].

Before going into detail about attempts to mitigate the inherent weaknesses, the as-built conditions must be addressed. Due to the rapid cooling rates in LPBF, the α-Al is present in a fine-grained structure and is also highly oversaturated [3]. Therefore, right after the scan track or layer is fabricated, the strength is governed by solid solution strengthening and possibly grain refinement. It is believed that subsequent heat input during the fabrication of neighboring scan tracks and subsequent layers starts the artificial aging process by first forming clusters of magnesium and silicon atoms. The stoichiometry of these clusters depends on the temperature during their formation and has a strong impact on the necessary duration of the formation of strength relevant intermetallic phases in Al–Si–Mg alloys [15,16,17,18]. From cast Al–Si–Mg it is known that clusters formed at elevated temperatures pose a beneficial stoichiometry and therefore enable a more effective precipitation hardening, which is utilized in direct-aging treatments [19,20]. Given that the LPBF fabrication takes place in a heated environment, in addition to the cluster formation, an artificial aging of already fabricated regions occurs whilst the component is still being fabricated. As a result, the prevailing hardening effect in the fabricated component may be both solid solution strengthening and precipitation hardening, with precipitation hardening being the dominant strengthening mechanism in age-hardenable Al–Si–Mg alloys [8,21]. Atom probe studies were recently utilized to determine the stoichiometry of the clusters formed in early stages of the aging sequence [15,16,22]. It was found that the stoichiometry of the clusters depended on the temperature of their formation. If aging at elevated temperatures was performed immediately after solution annealing, the stoichiometry of the clusters favored the precipitation of strength relevant β″-precipitates. The peak aged condition can therefore be achieved by shorter artificial aging [22].

Attempting to overcome the Si-segregations via heat treatments is by no means a new approach, and numerous attempts were made in the past, albeit with limited success. Secondary artificial aging and lower annealing temperatures were shown to promote enlarged Si-particles along the remolten areas [4]. Secondary artificial aging, however, can be utilized to remove the variance in the hardening mechanisms [23]. In cast Al–Si–Mg solution, annealing is performed to homogenize the microstructure and to remove segregations in the material composition, which are induced during slow cooling and alterations in the solubility levels. For cast parts, the solution annealing is performed until a complete homogenization is achieved. In order to achieve a similarly stable microstructure at temperatures above 500 °C, it was found that Al–Si–Mg fabricated by LPBF required a much longer solution annealing time [24]. However, such a treatment drastically transforms the microstructure, and in most cases, even after rapid quenching and artificial aging, the initial hardness and strength cannot be reached again [25,26,27]. On the positive side, high cycle fatigue performance was greatly enhanced [28,29]. None of the studies returned a conclusive statement regarding an ideal post-heat treatment for Al–Si–Mg fabricated by LPBF.

For this study, the aim was to resemble the cluster formation at elevated temperatures to achieve a best case scenario after a solution annealing step. The materials of choice were AlSi7Mg and AlSi10Mg. For reference purposes, sand- and die-cast samples were tested as well. First trials resulted in other than expected responses of the LPBF fabricated Al–Si–Mg to the quenching rates established for cast Al–Si–Mg, and therefore the investigation was extended to capture microstructural development at varying solution annealing times and the impact of quenching rates on the precipitation hardening capabilities.

## 2. Methodology

### 2.1. LPBF Samples

Samples were fabricated in two batches, one batch with AlSi7Mg0.3 powder (supplied by LPW Technology, Cheshire, UK) and the second batch with AlSi10Mg0.3 powder (supplied by SLM Solutions AG, Lübeck, Germany). An SLM 280HL machine (SLM Solutions AG, Lübeck, Germany), equipped with a 400 W Yb-fiber-laser (Model YLR-400-WC, IPG Photonics Corporation, Oxford, MA, USA) was utilized for fabrication. A detailed listing of the irradiation parameter sets and scan strategy settings is provided in Table 1.

**Figure 2:**
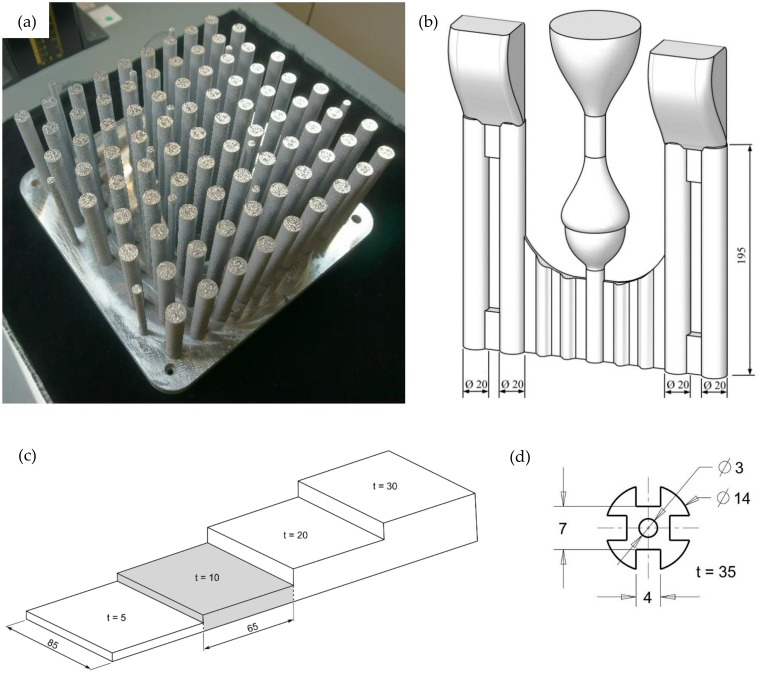
(**a**) Laser powder-bed fusion (LPBF) samples on substrate plate; (**b**) sand-cast assembly; (**c**) die-cast geometry; (**d**) modified sample geometry for improved quenching rates in the induction furnace.

Two kinds of samples were fabricated: Small cylindrical specimens with diameter 7 mm for dilatometer tests as well as larger cylindrical specimens with diameter 14 mm. Both sample types were built in a 90° configuration parallel to the built direction and had a total length of 100 mm (Figure 2a).

### 2.2. Cast Samples

The sand-cast samples were produced by Georg Fischer AG (Schaffhausen, Switzerland) from conventional AlSi7Mg, with strontium added for refinement purposes. Hydral 40 (Vesuvius plc, London, UK) was used as a gassing agent. Samples were cast as cylindrical bars with a diameter of 20 mm and a total length of 195 mm (Figure 2b). Die-cast samples were cast by Georg Fischer AG (Switzerland) from conventional AlSi7Mg. The melt was refined with strontium, and AlTi_5_B_1_ was added for grain refinement. Casting was performed with a preheated mold (330 °C) [30]. Only the 85 × 65 × 10 mm^3^ sections were utilized in this study (shaded in Figure 2c).

### 2.3. Sample Nomenclature

LPBF samples are referred to as LPBF-AlSi7 and LPBF-AlSi10, respectively. Similarly, sand-cast samples are named as SC-AlSi7 and die-cast samples as CC-AlSi7.

### 2.4. Spark Emission Spectroscopy

The material compositions were determined on non-heat-treated samples of each batch. In order to avoid any deviations due to surface defects, the samples were ground and subsequently analyzed via spark emission spectrometry (Spectromaxx-LMX06, SPECTRO Analytical Instruments GmbH, Kleve, Germany).

### 2.5. Heat Treatment

**Figure 3:**
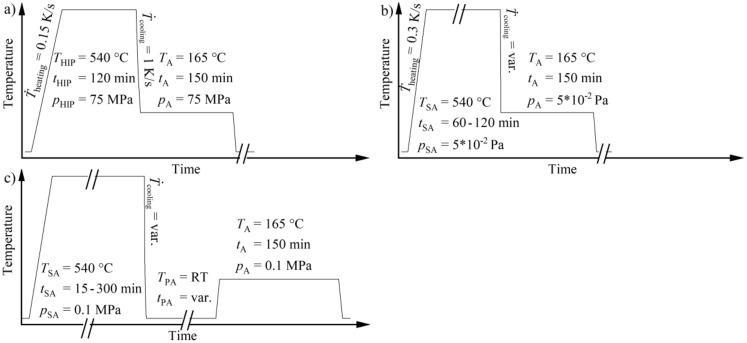
Schematic depiction of the applied heat treatments: (**a**) hot isostatic press; (**b**) dilatometer and induction furnace; (**c**) resistance furnace.

The microstructure and hardness of the aluminum alloys were evaluated in various conditions, which comprised heat treatments in a quenching and deformation dilatometer (Bähr DIL 805, TA Instruments, Hüllhorst, Germany), a resistance furnace (Naber, G. Mendheim GmbH, Munich, Germany), a self-developed vacuum induction furnace (described in detail in [31]) and a hot isostatic press (EPSI, Belgium). The hot isostatic press available offers the possibility of fast cooling and has been successfully employed to perform direct-aging heat treatments on aluminum cast alloys during hot isostatic pressing [19,20]. The direct-aging approach, which immediately continues with the artificial aging after quenching from solution annealing temperature, was used for all heat treatments, with the exception of the treatments done in the resistance furnace. The latter followed the traditional approach of removing the samples from the furnace and quenching in a cooling medium, which is followed by an artificial aging step after varying dwell times at room temperature. A complete listing of the heat treatment procedures with the introduction of the nomenclature is provided in Table 2, whereas Figure 3 shows sketches of the heat treatment schedules.

Samples were machined to suit the employed equipment, the details on the geometries are listed in Table 3. To enhance the achievable quenching rates, a second sample geometry with an increased surface area to volume ratio was utilized in the induction oven (Figure 2d).

### 2.6. Hardness Evaluation

Hardness measurements were performed with two hardness testing machines. An EMCO M4U-025 (Maier Ges.m.b., Hallein, Austria) was utilized for Brinell hardness (HBW2.5/62.5) measurements and a Dia Testor 2 Rc (Otto Wolpert-Werke GmbH, Ludwigshafen, Germany) for Vickers hardness (HV10) measurements (for comparison purposes, the conversion tables put down in the ASTM E140-07 standard [32] were utilized.). Due to size restrictions on some samples, smaller Vickers indentations were chosen to allow for multiple measurements with sufficient distance in between adjacent indentations.

### 2.7. Micro Sections

The preparation for the microstructural characterization involved a two stage grinding, followed by a three stage polishing procedure. Following first inspection, the surfaces were etched with a 5% molybdic acid. Microstructure analyses were performed with a light optical microscope (Aristomet, Ernst Leitz Wetzlar GmbH, Wetzlar, Germany).

### 2.8. Porosity Measurements

The weight of samples was determined on a Sartorius Research R300 S scale in air and in water, utilizing a setup based on Archimedes’ principle. It should be noted that the water contained traces of additives to reduce the surface tension of the fluid to enhance the reliability of the measurements. The obtained sample densities were compared with the calculated theoretical density of the respective alloy to obtain the residual porosity.

### 2.9. Atom Probe Tomography

Atomic resolution in three dimensions encompassing the entire periodic system characterization and quantification is offered by the atom probe tomography (APT) technique. With APT the clustering of silicon and magnesium atoms in the early stages of the precipitation sequence of intermetallic phases was studied. For specimen preparation, a sharp tip with less than 100 nm diameter is needed. Additively, manufactured Al–Si–Mg alloys possess Si-segregations, which mostly appear in the bonding areas between single scan tracks and subsequent layers. Since the eutectic silicon region etches differently than the matrix, an irregular shape of the tip is obtained if electrolytic polishing methods are applied. In order to achieve a regular shape of the APT tip and to ensure a defined distance to the top of the samples, the lift-out method using scanning electron microscope with a focused ion beam was used, according to [33]. By applying a low voltage, cleaning gallium implantation in the specimen was reduced. The samples were lifted out from a plane surface with a distance of 2 mm measured from the top of the standing LPBF aluminum cylinders; see Figure 2a. The measurements were done in voltage mode at 40 kHz and 200 kHz and 20% evaporation rate, using the LEAP 3000× HR system. The software IVA3.6 from Cameca (Gennevilliers, France) was used for data evaluation.

## 3. Results

### 3.1. Chemical Compositions

In comparison to the cast samples, an increased silicon content was noted for the LPBF samples; see Table 4. Slight fluctuations in the magnesium content were evident, ranging from 0.28 wt.% for CC-AlSi7 to 0.37 wt.% for LPBF-AlSi10.

### 3.2. Hardness

**Figure 4:**
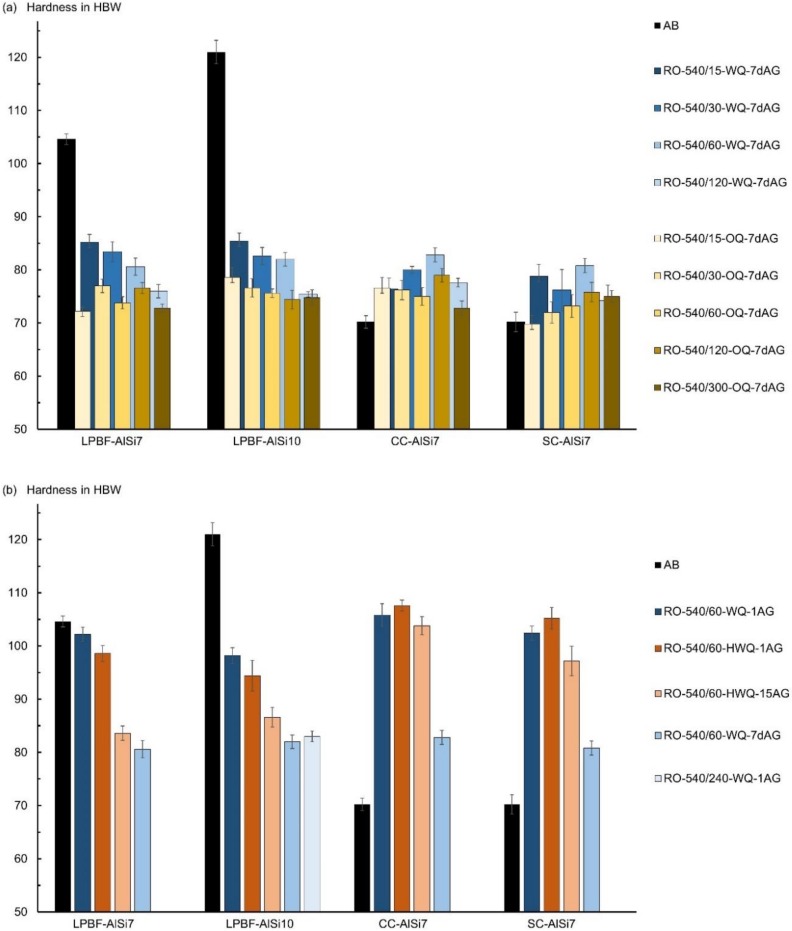
Hardness results for cast and laser powder-bed-fused samples in as-built (AB) and heat treated conditions; all results refer to heat treatments in the resistance furnace (RO) and were artificially aged (AG) for 150 min at 165 °C. Oil quenched (OQ) and water quenched (WQ) results at varying solution annealing times are depicted in (**a**); hot water quenched (HWQ) results and varying dwell times at room temperature are shown in (**b**). The utilized nomenclature is explained in detail in Table 2.

Test series employing conventional heat treatment in a resistance furnace, with a dwell time in between heat treatment steps (simulated by a 7 d delay between quenching and artificial aging) are depicted in Figure 4a. The hardness of die-cast (CC) and sand-cast (SC) samples stabilized at around 60–120 min of solution annealing time, after which a further increase in solution annealing time resulted in no further increase in hardness. Oil quenched samples seemed to obtain their highest hardness results after 120 min of solution annealing, whereas the maximum hardness of water quenched samples was observed after 60 min. Water quenching resulted in the highest hardness after artificial aging of all sample types, delivering better or on par results compared to quenching in oil. To study a possible effect of fluctuations in water temperature, quenching in water of 20 °C and 80 °C was done, showing however only minor deviations within the margins of error, included in Figure 4b. The initial hardness of CC and SC samples was raised from 70 HBW to above 80 HBW.

In contrast to the behavior of the cast samples, the hardness of the LPBF samples decreased significantly compared to their respective hardness in the as-built (AB) condition (Figure 4a). With increasing solution annealing times, the hardness after artificial aging decreased even further, facing a constant value of around 75 HBW past 120 min of solution annealing. Initial hardness differences between LPBF-AlSi7 and LPBF-AlSi10 remained mildly present after 15 min of solution annealing time but vanished at the 30 min mark. Immediate aging (~1 min delay between quenching and artificial aging) led to a drastically increased hardness, in the range from 98–106 HBW. The dwell time influence for short delays appeared to be more pronounced for LPBF samples than it was for the SC and CC samples. In comparison to the immediately aged samples, a dwell time of 15 min at room temperature resulted in a substantial decrease in hardness (8–15 HBW) for LPBF samples, whilst cast samples decreased by 4–8 HBW (Figure 4b). These deviations vanished after longer dwell times; samples aged after a 7 d dwell time at room temperature showed consistent hardness and a similar hardness reduction compared to the immediately aged condition. These results show that immediate aging is applicable and beneficial on LPBF samples. LPBF-AlSi7 samples reacquired their initial hardness, whereas in LPBF-AlSi10 samples it stayed 20 HBW below their hardness in as-built condition. Cast samples, however, exhibited a more pronounced increase in hardness, due to immediate aging, than LPBF samples.

Performing the HIP-540/120-1-DA direct-aging heat treatment in the hot isostatic press returned unexpected results. There, the solution annealing time was set to 120 min due to the slower diffusion of alloying elements at increased pressure. For the maximum quenching rate of 1 K/s, the SC-AlSi7 samples acquired a hardness of around 80 HBW, which is comparable to that of conventionally heat treated results, such as water quenched and artificially aged after longer dwell times (RO-540/120-WQ-7dAG). Large differences were evident for LPBF-AlSi10, whose hardness dropped to 54 ± 1.9 HBW, resembling the hardness of AlSi10 in the absence of precipitation hardening.

**Figure 5:**
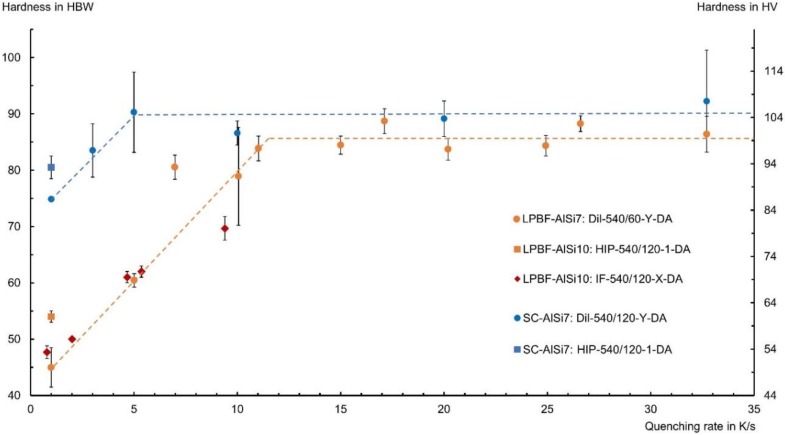
Hardness in dependence of the quenching rate after solution annealing followed by direct aging. The dashed levelling lines symbolize the quenching rate dependency of LPBF and SC samples. Denoted quenching rates (*x*-axis) refer to the average cooling rate between 535 °C and 200 °C.

Studies with the dilatometer confirmed that the LPBF samples require faster quenching rates than the SC reference samples (Figure 5). While the hardness of SC-AlSi7 remained constant above an average quenching rate of 5 K/s under vacuum condition, LPBF-AlSi7 required about 11–15 K/s to achieve similar hardness values. These results of the dilatometer tests are in agreement with those obtained in the induction furnace for the series LPBF-AlSi10. In short, LPBF samples showed no noteworthy precipitation hardening below a quenching rate of 2 K/s. For higher quenching rates, a linear increase of the hardness was observed until a hardness of about 100 HV was reached. Compared to the behavior of the HIP-540/120-1-DA results, both LPBF-AlSi7 and SC-AlSi7 exhibited a higher hardness, which indicates an influence of decreased diffusion rates at increased pressure.

### 3.3. Micro Sections

**Figure 6:**
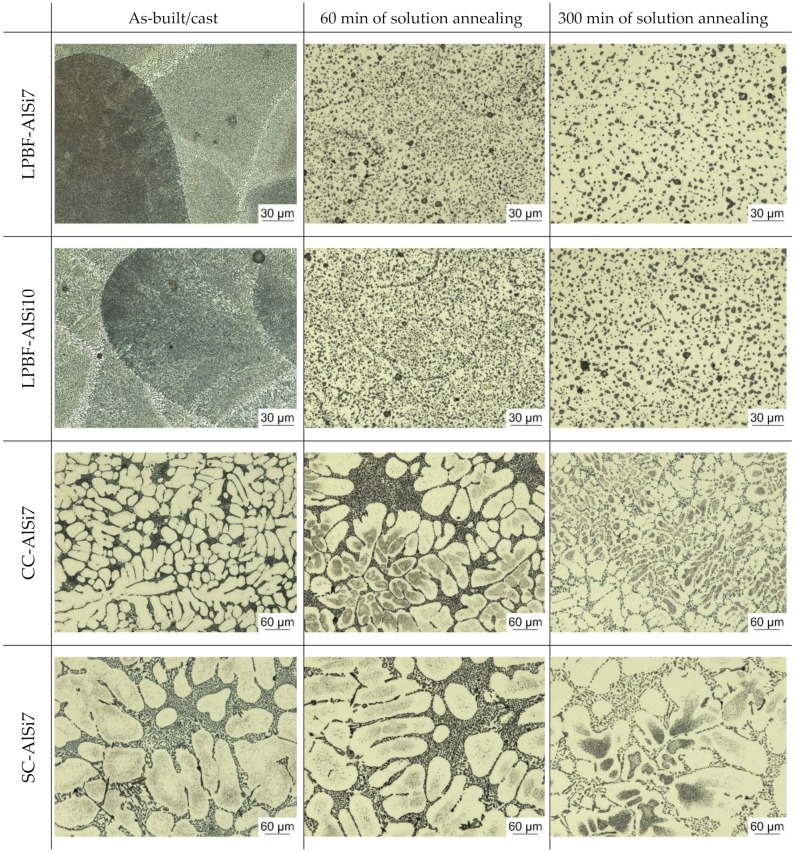
Comparison of microstructural evolution during heat treatment for laser powder-bed fused and cast samples in their as-built and two solution annealed conditions (60 min and 300 min at 540 °C), followed by rapid quenching.

**Figure 7:**
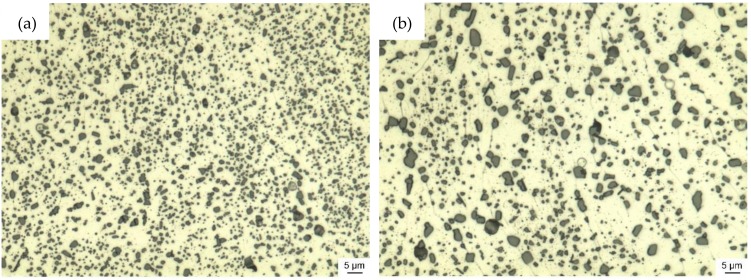
Micro sections of LPBF-AlSi7 samples for (**a**) 15 min and (**b**) 60 min solution annealing, followed by rapid quenching, illustrating coarsening for α-Al and Si particles.

The appearance of the microstructure for the LPBF samples in their as-built (AB) condition is closely linked to the scan track pattern employed during fabrication (Figure 6). During solution annealing, the finely dispersed Si-segregations coarsened and reduced the visibility of this interlaced pattern. The dominance of the formation of Si-segregations along the scan track boundaries was clearly observable on the 60 min solution annealed samples. From 60 to 300 min solution annealing time, the coarsening continued, while the dominant growth came to a hold at around 120 min. Magnified micro sections images illustrating the coarsening of both α-Al and Si particles are depicted in Figure 7.

### 3.4. Porosity

LPBF samples exhibited a higher amount of porosity than cast samples. In the as-built condition the porosity (Porosity was calculated via the density obtained in the Archimedes method, divided by the theoretical density of the alloy) was 0.3% in LPBF-AlSi7 and 0.7% in LPBF-AlSi10 samples. CC-AlSi7 samples had the lowest porosity with 0.001%, followed by SC-AlSi7 samples with 0.17%. The residual porosity in cast samples is caused by shrinking, and thus voids are mostly empty. In contrast to this, pores in LPBF-AlSi samples are likely to contain either the inert gas utilized during the fabrication process or hydrogen, which stems from moisture in the powder. During heat treatment, these gas-filled pores tend to expand, leading to an increase in porosity. This expansion was observed to take place within the first 30 min of solution annealing; samples from 30 min to 300 min of solution annealing exhibited similar porosities. The porosity of cast samples increased mildly to 0.2% in CC-AlSi7 and 0.4% in SC-AlSi7 samples. A significant increase in porosity was observed for the LPBF samples; the porosity rose to 2.9% in LPBF-AlSi7 and 2.0% in LPBF-AlSi10. Fluctuations between sample series in the induction furnace and resistance furnace were within margins of error. Hot isostatic pressing was able to improve the porosity in both cases. No remaining porosity was found in hot isostatic pressed SC-AlSi7 samples, and the porosity in LPBF-AlSi10 samples was lowered to 0.02%.

### 3.5. Atom Probe Tomography

**Figure 8:**
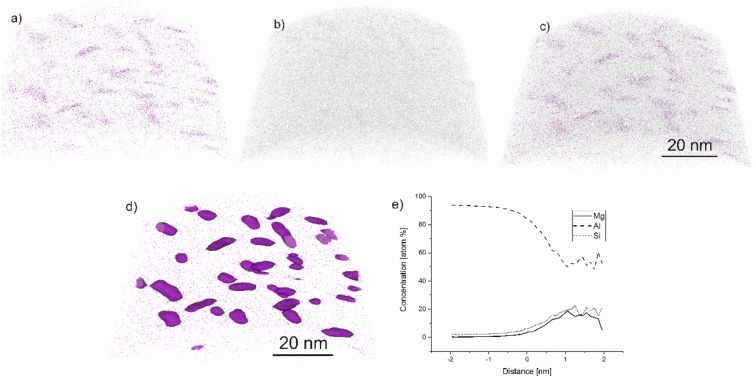
Atom distribution on LPBF-AlSi10 in the as-built condition for Mg (**a**) and Si (**b**) and both elements together (**c**). (**d**) shows the distribution of the Mg atoms with 3 at.% isosurface; (**e**) is the proxigram, showing the chemical composition for all the precipitated phases; the distance (*x*-axis) refers to the distance relative to the interface/isosurface. Zero indicates the interface itself, negative *x*-values the chemical composition outside (matrix) and positive *x*-values inside (cluster) the interface.

Figure 8 depicts the results of the atom probe tomography. Figure 8a shows the distribution of Mg atoms, while the distribution of Si Atoms is depicted in Figure 8b. The concentration isosurface for Mg is given in Figure 8d for a concentration of 3 at.% of Mg. These illustrate that the LPBF Al–Si–Mg alloy possesses clusters that contain both Si and Mg atoms. These clusters are needle shape with a diameter of about 2 to 5 nm and a length of 10 to 15 nm. Figure 8e is a proxigram, which illustrates the concentration of Al, Si and Mg atoms in the aluminum matrix (distance −2 to 0.5 nm) and the clusters (distance 0.5 to 2 nm).

## 4. Discussion

Age-hardenable aluminum alloys processed by LPBF require faster quenching after solution annealing than their cast counterparts. A quenching rate of 1 K/s was insufficient for LPBF-AlSi10 during the combined hot isostatic pressing heat treatment to achieve an oversaturation, and as a result almost no precipitation hardening was possible. The measured hardness of 54 HBW is a typical hardness after solution annealing and quenching. The sample series RO-540/120-OQ-7dAG ranged between 56–61 HBW after quenching, prior to natural and artificial aging. LPBF samples were shown to require a cooling rate above 10 K/s in the temperature range between 535 °C and 200 °C under vacuum condition to allow for a sufficient precipitation hardening. A possible explanation for this could be the finer microstructure of the LPBF samples and thus the shorter diffusion paths, which lead to a faster depletion of the dissolved elements in the α-Al. Slight deviations between the dilatometer test series and those of the resistance furnace are evident, which may be attributed to the different sample sizes and the homogeneity of the temperature distribution during the heat treatment.

While T6-like heat treatments are widely promoted as homogenizing treatments for LPBF-AlSi, it was shown that in the best case scenario the high initial hardness of the as-built condition can be restored. However, 100 HBW was the maximum hardness reached, which is still far below the hardness of the LPBF-AlSi10 samples in their as-built condition (120 HBW). While the quenching rate above a certain value appears to become irrelevant, it is important to remember that due to the small melt pool relative to the size of the part or substrate beneath, the self-quenching effect in LPBF is much higher than the achievable quenching rates during heat treatments, and thus a further increase in hardness for much higher quenching rates cannot be ruled out [7,34,35]. Considering that the oversaturation reached in LPBF may be much higher than the oversaturation achieved during our heat treatments, the increased solid solution strengthening and precipitation hardening could explain the offset by 20 HBW. This assumption is supported by the particle area measurements reported by Li, et al. [36]. The total area occupied by Si particles increased by around 14% within the first 2 h of solution annealing. With the maximum solubility of 1.65 wt.% for Si in α-Al, this increase cannot be explained. Therefore, an increased solubility or very fine-dispersed Si-particles, which likely remained hidden in the scanning electron microscope images, may be the cause. Either factor would enhance the hardness in the as-built condition and explain the significant hardness deviations seen in LPBF-AlSi10.

Atom probe tomography was performed for material regions located at a distance of 2 mm below the top face of a standing LPBF sample; see Figure 2a. The results of the atom probe tomography demonstrated that the microstructure near the top of the samples contained clusters of Si and Mg atoms. When comparing the size and shape of these clusters to relevant literature data [22], it can be noted that the clustering sequence was at least in an intermediate stage. Even though the top of the as-built material was not artificially aged for an extended period during fabrication, the region nevertheless exhibited significant atom clustering. The tendency to form such clusters is probably driven by the heat stored in the already built structure as well as in the surrounding powder bed. Due to the low heat dissipation through the powder-bed, which results from the large interface area between the powder particles and the process gas, the heat dissipation to the sides of the build chamber is low. In addition, the high number of alloying elements lowers the thermal conductivity of aluminum alloys [34,37,38]. These two effects delay the final cooldown of the built samples and allow for a sufficiently long time to start precipitation hardening. The temperature over time schedule after the LPBF process corresponds to a direct-aging treatment, which was shown to shorten the time for clustering of Si and Mg atoms in age-hardenable Al–Si–Mg alloys. The beneficial clustering of Si and Mg atoms is another factor contributing to the outstanding materials strength of LPBF-AlSi in the as-built condition.

**Figure 9:**
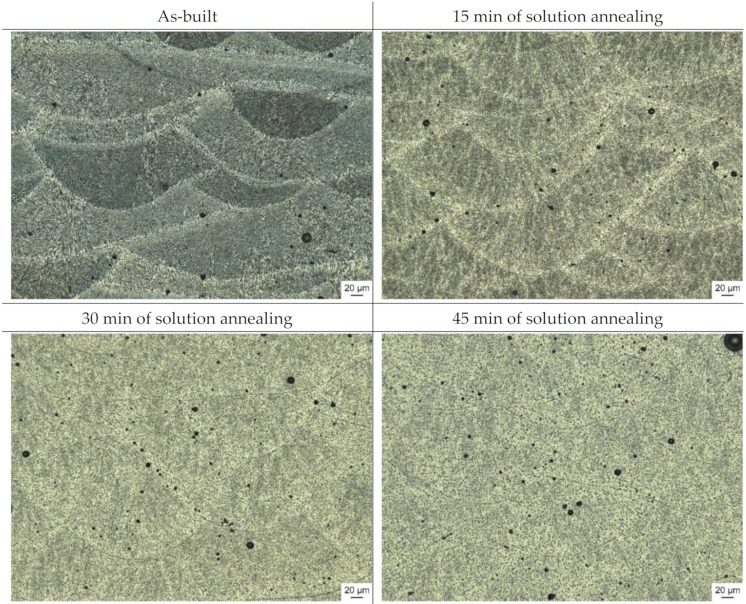
Development of the microstructure of laser powder-bed fused AlSi10Mg samples during solution annealing at 540 °C. The gradual transformation from the distinct layered structure to a more homogeneous structure is shown.

The LPBF microstructure undergoes a complete transformation within the first 45 to 60 min of solution annealing, during which the α-Al grains coarsened significantly (Figure 7 and Figure 9). This coarsening is a result of the absence of the finely dispersed Si-particles along the α-Al boundaries, which agglomerated to enlarged Si particles and were preferably located at the heat affected zones in the material at as-built condition. Lowering the solution annealing temperature to slow down the coarsening process was reported to be detrimental regarding achievable material strength and hardness [39]. 

## 5. Conclusions

LPBF-AlSi samples were heat treated, and their hardness response to solution annealing times and quenching rates was examined. LPBF-AlSi requires longer solution annealing times of up to 2 h for the microstructure to stabilize. During this time the α-Al grains coarsen significantly, and the finely dispersed Si-segregations agglomerate to larger Si-precipitates, preferably located at the heat affected zones in the material at as-built condition. While these larger Si particles mostly eliminate the directional weakening the Si-segregations cause in the as-built condition, they are too large to contribute to the material’s strength. Significant differences in the required quenching rates to achieve an oversaturated condition of dissolved silicon and magnesium atoms in the aluminum matrix, which is necessary for precipitation hardening, were observed. While cast reference material required a quenching rate of about 5 K/s to achieve appreciable age-hardening under vacuum condition, the same aluminum alloy required a quenching rate of 11–15 K/s if manufactured by means of LPBF process. This large deviation may be attributed to the finer microstructure and thus shorter diffusion paths in the microstructure of LPBF fabricated material, whereby achieving a metastable oversaturation for precipitation hardening is more challenging. 

The best post-heat treatment scenario was able to restore the initial hardness value. However, this was only the case for the lower alloyed AlSi7Mg alloy; for AlSi10Mg a gap of about 20 HBW remained, with its initial hardness after fabrication being superior. The reasons for this are believed to be twofold. The fine structures in both the α-Al and the Si particles, which at this scale could be beneficial for the material strength, coarsen significantly within the first 30 min of solution annealing. This effect is coupled with the significantly lower quenching rates in post-heat treatments compared to the cooling rates in LPBF fabrication process and thus result in a less effective precipitation hardening.

## Figures and Tables

**Table 1:** Parameter sets utilized for fabrication.

	Scan Speed (mm/s)	Laser Power (W)	Hatch Distance (mm)	Scan Vector Length (mm)	Rotation Angle Increment (°)
**AlSi7Mg0.3**					
Core	1050	350	0.17	10	67
Support	900	350	—	—	—
**AlSi10Mg0.3**					
Core	1150	350	0.17	10	67
Support	900	350	—	—	—
**Common**					
Preheating temperature set to 200 °C					
Layer thickness of 50 μm					
Argon environment					
Contour irradiation and limitation window deactivated					

**Table 2:** Detailed explanation of applied heat treatments and nomenclature.

Abbreviation	Apparatus	Treatment
AB	None	As-built condition without any additional treatment being applied
HIP-540/120-1-DA	Hot isostatic press	Solution annealed at 540 °C for 120 min, quenched at a rate of 1 K/s to 165 °C, aged at 165 °C for 150 min; treatment performed in argon at a pressure of 75 MPa
Dil-540/X-Y-DA	Dilatometer	Solution annealed at 540 °C for *X* min, quenched at a rate of *Y* K/s to 165 °C, aged at 16 5 °C for 150 min; treatment performed under vacuum
IF-540/2-X-DA	Induction furnace	Solution annealed at 540 °C for 120 min, quenched at a rate of X K/s to 165 °C, aged at 165 °C for 150 min; treatment performed under vacuum
RO-540/X-Y-ZAG	Resistance furnace	Solution annealed at 540 °C for *X* min, quenched with *Y* = oil quenched (OQ; oil at 20 °C); water quenched (WQ; water at 20 °C); hot water quenched (HWQ; water at 80 °C), followed by a dwell time at room temperature of *Z* = 1 (1 min, immediate aging); 15 (15 min at RT); 7 d (7 days at RT), aged at 165 °C for 150 min; treatment performed in air

**Table 3:** Summary of the machined sample geometries for different heat treatment apparatuses.

Apparatus	Sample Geometry	Quenching Rate in the Temperature Range 540 to 200 °C
Dilatometer	diameter of 5 mm and 8 mm in length	up to ~33 K/s
Resistance furnace	8 mm thick slices of cylindrical samples (LPBF, sand-cast—SC); 40 × 30 × 8 mm^3^ (die-cast—CC)	depending on quenching media
Hot isostatic press	diameter of 16 mm and 75 mm in length	up to ~1 K/s
Induction furnace	diameter of 14 mm and 35 mm in length	up to ~5 K/s
	diameter of 14 mm and 35 mm in length, with enlarged surface area as depicted in Figure 2d	up to ~9 K/s

**Table 4:** Chemical compositions obtained via spark emission spectrometry on laser powder-bed fused and cast samples; all values in wt.%.

	Laser Powder-Bed Fused Samples			
	LPBF-AlSi7Mg0.3		LPBF-AlSi10Mg0.3	
	Average	Deviation	Average	Deviation
Si	7.947	0.343	12.483	1.180
Fe	0.134	0.026	0.205	0.006
Cu	0.021	0.002	0.004	0.000
Mn	0.008	0.005	0.015	0.005
Mg	0.373	0.017	0.297	0.122
Ni	0.023	0.007	0.021	0.002
Zn	0.125	0.026	0.048	0.009
Ti	0.012	0.012	0.034	0.006
Sr	0.001	0.001	0.003	0.000
Al	balance		balance	
	**Cast samples**			
	**Die-Cast AlSi7Mg0.3**		**Sand-Cast AlSi7Mg0.3**	
	Average	Deviation	Average	Deviation
Si	6.883	0.361	7.047	0.281
Fe	0.098	0.010	0.151	0.017
Cu	0.007	0.001	0.038	0.008
Mn	0.007	0.001	0.027	0.001
Mg	0.279	0.045	0.352	0.047
Ni	0.006	0.003	0.006	0.002
Zn	0.012	0.008	0.068	0.020
Ti	0.116	0.019	0.106	0.004
Sr	0.014	0.004	0.019	0.002
Al	balance		balance	

## References

[1] Hitzler L., Merkel M., Hall W., Öchsner A. (2018). A review of metal fabricated with laser- and powder-bed based additive manufacturing techniques: Process, nomenclature, materials, achievable properties, and its utilization in the medical sector. Adv. Eng. Mater..

[2] DebRoy T., Wei H.L., Zuback J.S., Mukherjee T., Elmer J.W., Milewski J.O., Beese A.M., Wilson-Heid A., De A., Zhang W. (2018). Additive manufacturing of metallic components – process, structure and properties. Prog. Mater. Sci..

[3] Aboulkhair N.T., Simonelli M., Parry L., Ashcroft I., Tuck C., Hague R. (2019). 3D printing of Aluminium alloys: Additive Manufacturing of Aluminium alloys using selective laser melting. Prog. Mater. Sci..

[4] Prashanth K.G., Scudino S., Klauss H.J., Surreddi K.B., Löber L., Wang Z., Chaubey A.K., Kühn U., Eckert J. (2014). Microstructure and mechanical properties of Al–12Si produced by selective laser melting: Effect of heat treatment. Mater. Sci. Eng. A.

[5] Hitzler L., Hirsch J., Heine B., Merkel M., Hall W., Öchsner A. (2017). On the anisotropic mechanical properties of selective laser melted stainless steel. Materials.

[6] Hitzler L., Hirsch J., Tomas J., Merkel M., Hall W., Öchsner A. (2019). In-plane anisotropy of selective laser melted stainless steel: The importance of the rotation angle increment and the limitation window. Proc. Inst. Mech. Eng. Part L.

[7] Prashanth K.G., Eckert J. (2017). Formation of metastable cellular microstructures in selective laser melted alloys. J. Alloys Compd..

[8] Hitzler L., Janousch C., Schanz J., Merkel M., Heine B., Mack F., Hall W., Öchsner A. (2017). Direction and location dependency of selective laser melted AlSi10Mg specimens. J. Mater. Process. Technol..

[9] Hitzler L., Schoch N., Heine B., Merkel M., Hall W., Öchsner A. (2018). Compressive behaviour of additively manufactured AlSi10Mg. Mat. Wiss. Werkstofftech..

[10] Hitzler L., Hirsch J., Schanz J., Heine B., Merkel M., Hall W., Öchsner A. (2019). Fracture toughness of selective laser melted AlSi10Mg. Proc. Inst. Mech. Eng. Part L.

[11] Hitzler L., Sert E., Schuch E., Öchsner A., Merkel M., Heine B., Werner E. (2020). Fracture toughness of L-PBF fabricated aluminium-silicon: A quantitative study on the role of crack growth direction with respect to layering. Prog. Addit. Manuf..

[12] Aboulkhair N.T., Maskery I., Tuck C., Ashcroft I., Everitt N.M. (2016). The microstructure and mechanical properties of selectively laser melted AlSi10Mg: The effect of a conventional t6-like heat treatment. Mater. Sci. Eng. A.

[13] Hitzler L., Sert E., Merkel M., Öchsner A., Werner E. (2019). Fracture toughness and fatigue strength of selective laser melted aluminium-silicon: An overview. TMS 2019 148th Annual Meeting & Exhibition Supplemental Proceedings.

[14] Tang M., Pistorius P.C. (2017). Oxides, porosity and fatigue performance of AlSi10Mg parts produced by selective laser melting. Int. J. Fatigue.

[15] De Geuser F., Lefebvre W., Blavette D. (2006). 3D atom probe study of solute atoms clustering during natural ageing and pre-ageing of an Al-Mg-Si alloy. Philos. Mag. Lett..

[16] Serizawa A., Hirosawa S., Sato T. (2008). Three-dimensional atom probe characterization of nanoclusters responsible for multistep aging behavior of an Al-Mg-Si alloy. Metall. Mater. Trans. A.

[17] Murali S., Arunkumar Y., Chetty P.V.J., Raman K.S., Murthy K.S.S. (1997). The effect of preaging on the delayed aging of Al−7Si−0.3Mg. JOM.

[18] Hafenstein S., Werner E. (2018). Simultaneous hot isostatic pressing and solution annealing of aluminium cast alloys followed by instantaneous aging at elevated temperatures. IOP Conference Series: Materials Science and Engineering.

[19] Hafenstein S., Werner E. (2019). Direct aging of a hot isostatically pressed A356 aluminum cast alloy. Mater. Sci. Eng. A.

[20] Hafenstein S., Werner E. (2019). Pressure dependence of age-hardenability of aluminum cast alloys and coarsening of precipitates during hot isostatic pressing. Mater. Sci. Eng. A.

[21] Buchbinder D., Meiners W., Wissenbach K., Poprawe R. (2015). Selective laser melting of aluminum die-cast alloy—correlations between process parameters, solidification conditions, and resulting mechanical properties. J. Laser Appl..

[22] Sha G., Möller H., Stumpf W.E., Xia J.H., Govender G., Ringer S.P. (2012). Solute nanostructures and their strengthening effects in Al–7Si–0.6Mg alloy F357. Acta Mater..

[23] Sert E., Hitzler L., Heine B., Merkel M., Werner E., Öchsner A. (2019). Influence of heat treatments on the microstructure and hardness of additive manufactured AlSi10Mg samples. Pract. Metallogr..

[24] Aboulkhair N.T., Tuck C., Ashcroft I., Maskery I., Everitt N.M. (2015). On the precipitation hardening of selective laser melted AlSi10Mg. Metall. Mater. Trans. A.

[25] Tang M. (2017). Inclusions, Porosity, and Fatigue of AlSi10Mg Parts Produced by Selective Laser Melting. Ph.D. Thesis.

[26] Aboulkhair N.T. (2016). Additive Manufacture of an Aluminium Alloy: Processing, Microstructure, and Mechanical Properties. Ph.D. Thesis.

[27] Hitzler L., Charles A., Öchsner A. (2016). The influence of post-heat-treatments on the tensile strength and surface hardness of selective laser melted AlSi10Mg. Defect Diffus. Forum.

[28] Aboulkhair N.T., Maskery I., Tuck C., Ashcroft I., Everitt N.M. (2016). Improving the fatigue behaviour of a selectively laser melted aluminium alloy: Influence of heat treatment and surface quality. Mater. Des..

[29] Brandl E., Heckenberger U., Holzinger V., Buchbinder D. (2012). Additive manufactured AlSi10Mg samples using selective laser melting (SLM): Microstructure, high cycle fatigue, and fracture behavior. Mater. Des..

[30] Brummer M. (2013). Wärmebehandelndes heißisostatisches Pressen von Aluminiumgusslegierungen. Ph.D. Thesis.

[31] Hafenstein S. (2019). Heißisostatisches Pressen von Aluminiumgusslegierungen mit integrierter Wärmebehandlung.

[32] (2007). E140–12b: Standard Hardness conversion Tables for Metals Relationship Among Brinell Hardness, Vickers Hardness, Rockwell Hardness, Superficial Hardness, Knoop Hardness and Scleroscope Hardness.

[33] Saxey D.W., Cairney J.M., McGrouther D., Honma T., Ringer S.P. (2007). Atom probe specimen fabrication methods using a dual fib/sem. Ultramicroscopy.

[34] Kimura T., Nakamoto T., Mizuno M., Araki H. (2017). Effect of silicon content on densification, mechanical and thermal properties of al-xsi binary alloys fabricated using selective laser melting. Mater. Sci. Eng. A.

[35] Jägle E.A., Sheng Z., Wu L., Lu L., Risse J., Weisheit A., Raabe D. (2016). Precipitation reactions in age-hardenable alloys during laser additive manufacturing. JOM.

[36] Li X.P., Wang X.J., Saunders M., Suvorova A., Zhang L.C., Liu Y.J., Fang M.H., Huang Z.H., Sercombe T.B. (2015). A selective laser melting and solution heat treatment refined Al–12Si alloy with a controllable ultrafine eutectic microstructure and 25% tensile ductility. Acta Mater..

[37] Hafenstein S., Brummer M., Ahlfors M., Werner E. (2016). Combined hot isostatic pressing and heat treatment of aluminum A356 cast alloys. HTM J. Heat Treat. Mater..

[38] Hafenstein S., Werner E., Wilzer J., Theisen W., Weber S., Sunderkötter C., Bachmann M. (2015). Influence of temperature and tempering conditions on thermal conductivity of hot work tool steels for hot stamping applications. Steel Res. Int..

[39] Iturrioz A., Gil E., Petite M.M., Garciandia F., Mancisidor A.M., San Sebastian M. (2018). Selective laser melting of alsi10mg alloy: Influence of heat treatment condition on mechanical properties and microstructure. Weld. World.

